# Structural and molecular basis for urea recognition by *Prochlorococcus*

**DOI:** 10.1016/j.jbc.2023.104958

**Published:** 2023-06-26

**Authors:** Chen Wang, Wen-jing Zhu, Hai-tao Ding, Ning-hua Liu, Hai-yan Cao, Chuan-lei Suo, Ze-kun Liu, Yi Zhang, Mei-ling Sun, Hui-hui Fu, Chun-yang Li, Xiu-lan Chen, Yu-Zhong Zhang, Peng Wang

**Affiliations:** 1 MOE Key Laboratory of Evolution and Marine Biodiversity, Frontiers Science Center for Deep Ocean Multispheres and Earth System & College of Marine Life Sciences, Ocean University of China, Qingdao, China; 2 State Key Laboratory of Microbial Technology, Shandong University, Qingdao, China; 3 Laboratory for Marine Biology and Biotechnology, Pilot National Laboratory for Marine Science and Technology, Qingdao, China; 4 Antarctic Great Wall Ecology National Observation and Research Station, Polar Research Institute of China, Shanghai, China; 5 Marine Biotechnology Research Center, State Key Laboratory of Microbial Technology, Shandong University, Qingdao, China

**Keywords:** urea transport, *Prochlorococcus*, ABC transporter, substrate-binding protein, mechanism

## Abstract

Nitrogen (N) is an essential element for microbial growth and metabolism. The growth and reproduction of microorganisms in more than 75% of areas of the ocean are limited by N. *Prochlorococcus* is numerically the most abundant photosynthetic organism on the planet. Urea is an important and efficient N source for *Prochlorococcus*. However, how *Prochlorococcus* recognizes and absorbs urea still remains unclear. *Prochlorococcus marinus* MIT 9313, a typical *Cyanobacteria*, contains an ABC-type transporter, UrtABCDE, which may account for the transport of urea. Here, we heterologously expressed and purified UrtA, the substrate-binding protein of UrtABCDE, detected its binding affinity toward urea, and further determined the crystal structure of the UrtA/urea complex. Molecular dynamics simulations indicated that UrtA can alternate between "open" and "closed" states for urea binding. Based on structural and biochemical analyses, the molecular mechanism for urea recognition and binding was proposed. When a urea molecule is bound, UrtA undergoes a state change from open to closed surrounding the urea molecule, and the urea molecule is further stabilized by the hydrogen bonds supported by the conserved residues around it. Moreover, bioinformatics analysis showed that ABC-type urea transporters are widespread in bacteria and probably share similar urea recognition and binding mechanisms as UrtA from *P. marinus* MIT 9313. Our study provides a better understanding of urea absorption and utilization in marine bacteria.

Nitrogen (N) is one of the fundamental elements of nucleic acid and protein and plays a vital role in the metabolism and energy transfer of organisms (1). The material circulation of the living system in much of the global ocean is limited by N (2). Dissolved organic nitrogen, one form of N in the ocean, has been confirmed to take an important role in controlling the structure, function, and species composition of the marine ecosystem (3).

Organisms generally preferentially take up reduced N, which avoids the costly reduction steps required to assimilate oxidized N (4). The reduced N, mostly in the form of urea and ammonium, often represents >50% of the total N in the marine environments, including the open oceans and coastal and estuarine systems (2). In the open oceans, urea is at the nanomolar levels, which is mainly derived from the metabolism of plankton and the mineralization of organic matter (1). It can be even found at concentrations of as high as 50 μM in some coastal ecosystems (5). In some estuarine and coastal areas, urea contributes to over half of all N required by phytoplankton (3). Therefore, urea is one of the most important marine dissolved organic nitrogen with the lowest molecular mass. As one of the main N sources, the contributions of urea to the regeneration productivity of the ocean are nonnegligible (6).

Various urea transporters (UTs) have been found in bacteria, animals, and plants (7, 8). They can be classified into two main types (7). Type Ⅰ includes the low-affinity membrane channel proteins transporting urea from high concentration to low concentration, and type Ⅱ contains the membrane proteins transporting urea against a concentration gradient by expending biological energy. Low-affinity membrane channel proteins are further grouped into three subtypes: the UT family transporter, the proton-gated urea channel/putative amide transporter (UreI/AmiS) family transporter, and the nonspecific water channel or major intrinsic protein (7, 9, 10, 11). Type Ⅱ, namely reverse concentration gradient membrane protein, contains two subtypes: DUR3 of the sodium-solute symporter family and the ATP-dependent ABC-type UT (12, 13). For low-affinity membrane channel proteins, the characters and urea recognition mechanisms have been studied in detail. In contrast, for the reverse concentration gradient membrane proteins, only putative DUR3/ABC-type transporters were proposed for urea transport based on some *in vivo* experiments (12, 14, 15, 16). Measurement of *in vitro* activity has not been performed, and molecular basis for urea binding is still unknown.

*Prochlorococcus* is both the smallest and numerically the most abundant photosynthesizing organism on the planet and can survival in nutrient-limited ocean regions (17, 18, 19). *Prochlorococcus* has been reported to contribute ∼50% of the photosynthetic biomass and net primary productivity in the oligotrophic marine regions from 40˚S to 40˚N, where nutrients, particularly the N source, essential for photosynthesis and cell growth, are scarce (17). The *urtABCDE* locus encoding a putative ABC-type UT (UrtABCDE) in *Anabaena* sp. PCC 7120 has been shown to be associated with high efficient urea transferring *in vivo*, which was also found in *Prochlorococcus marinus* MIT 9313, a typical *Prochlorococcus* strain (14). UrtABCDE consists of three parts: a substrate-binding protein (UrtA) located in the periplasm, two hydrophobic transmembrane domains (UrtB and UrtC), and two hydrophilic nucleotide-binding domains (UrtD and UrtE) (13, 20). UrtA from *P. marinus* MIT 9313 exhibits a sequence identity of 64.03% to UrtA from *Anabaena* sp. PCC 7120 (covering 98% of the full sequence). Although the function of UrtABCDE has been validated *in vivo*, it is still significant to investigate the structural basis and molecular mechanism of UrtABCDE for the efficient binding and transfer of urea.

To study how *Prochlorococcus* recognizes and absorbs urea, in this study, UrtA from *P. marinus* MIT 9313 was expressed in *Escherichia coli* BL21 (DE3) and purified, and the binding affinity of UrtA to urea was characterized. Furthermore, the crystal structure of UrtA/urea complex was determined. Based on structural analysis, biochemical validation, and molecular dynamics simulation (MDS), a molecular mechanism for the efficient recognition and binding of UrtA to urea was proposed. The distribution of ABC-type UTs and the universality of the recognition and binding mechanism of UrtA were investigated by bioinformatics analysis.

## Results and discussion

### Sequence analysis and purification of UrtA

**Figure 1:**
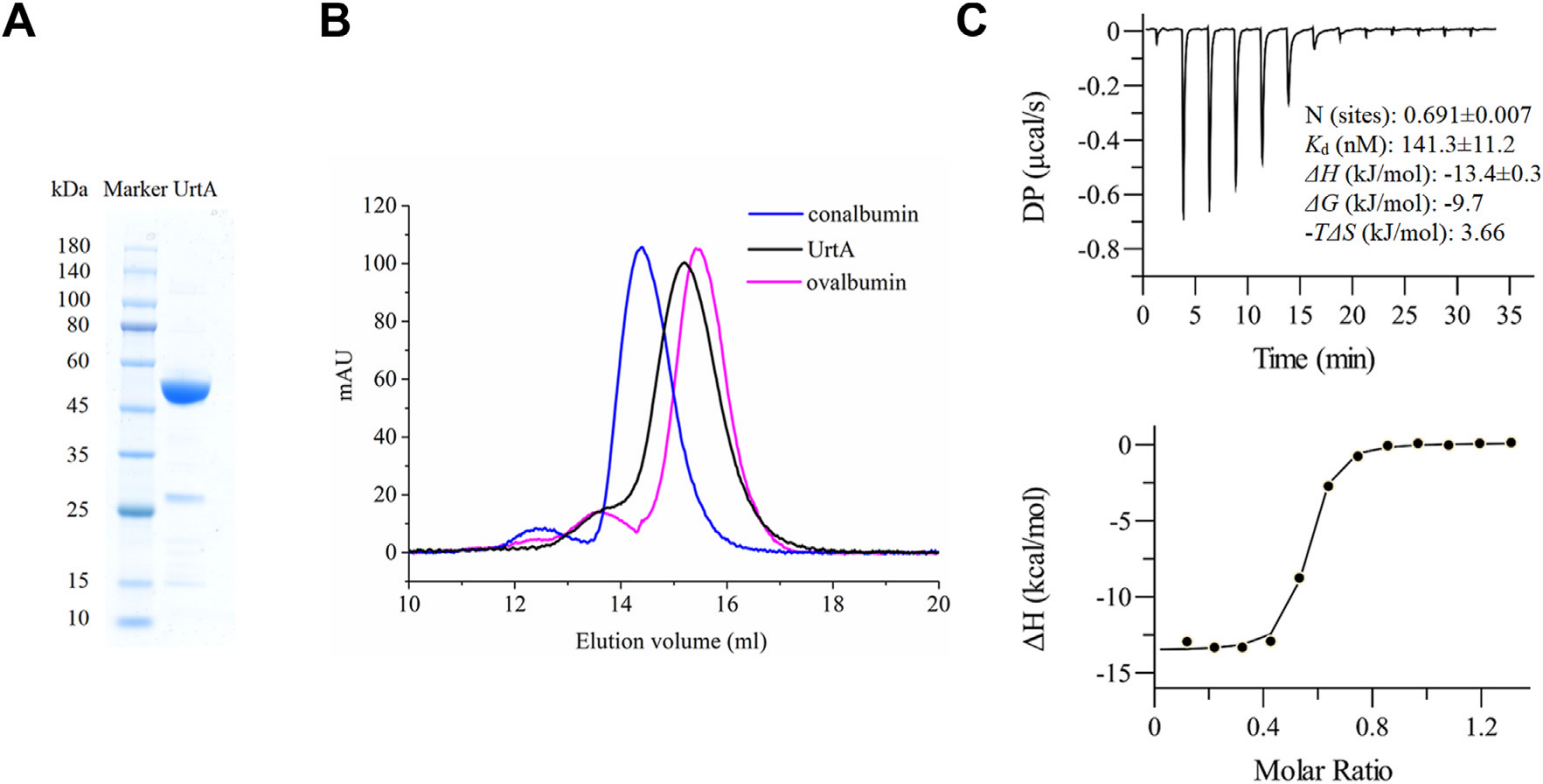
**Biochemical characterization of UrtA.***A*, SDS-PAGE analysis of the purified UrtA. *B*, gel filtration analysis of the form of UrtA in solution. Conalbumin (75 kDa; GE Healthcare) and ovalbumin (43 kDa; GE Healthcare) were used as the protein size markers. The predicted UrtA molecular mass of UrtA is 47 kDa. *C*, the affinity of UrtA to urea. ITC traces (*top*) and integrated binding isotherms (*bottom*) are shown. Δ*H*, change in enthalpy; Δ*G*, change in Gibbs free energy; *T*, temperature; Δ*S*, change in entropy. The fitting error of one experiment is shown, and the results of the other experimental repeats are provided in Table S1. ITC, isothermal titration calorimetry.

*urtA* of *P. marinus* MIT 9313 (GenBank accession No. BX548175) is 1296 bp in length, encoding a putative urea binding protein (UrtA) of 431 amino acid residues. The theoretical molecular weight of UrtA is 47,028.83 Da. UrtA contains a 24-residues signal peptide predicted by the SignalP 5.0 server. UrtA without the signal peptide was expressed in *E. coli* BL21 (DE3). The purified recombinant UrtA is approximately 50 kDa (Fig. 1*A*), consistent with its theoretical molecular weight. In addition, gel filtration analysis showed that the molecular weight of UrtA in solution is larger than 43 kDa and smaller than 75 kDa, indicating that UrtA presents as a monomer in solution (Fig. 1*B*).

### The binding affinity of UrtA to urea

To analyze the substrate binding affinity of UrtA, isothermal titration calorimetry (ITC) was used to detect the affinity of UrtA to urea. Compared to the other three types of UTs, UrtA exhibits a strong urea binding affinity, with a *K*_d_ of 141.3 ± 11.2 nM (Fig. 1*C*). The stoichiometry obtained was 0.691. Due to the presence of heterogeneous proteins, the determined concentration of the purified UrtA sample was higher than the actual concentration of UrtA in the sample, thereby resulting in a lower stoichiometry value. The actual stoichiometry value was likely close to 1. Previous studies have shown that similar substrate binding proteins bind one substrate molecule in one protein molecule (21, 22). The reduced Gibbs free energy (*ΔG*: −9.7 kJ/mol) calculated from *ΔH* (−13.4 kJ/mol) and *-TΔS* (3.66 kJ/mol) indicated a favorable exothermic binding process between UrtA and urea.

Different types of UTs exhibit different affinities to urea (Table 1). UreI from *Helicobacter pylori* was expressed heterologously and characterized as having a kinetic constant of ∼163 mM (10, 23). For DUR3s and UTs, no *in vitro* affinities were detected, but *in vivo* experiments revealed that the affinities to urea of DUR3s (3 μM for *Arabidopsis thaliana*, 21.9 μM for *Zea mays* roots, 31.8 μM for the ectomycorrhizal fungus *Paxillus involutus*) are always higher than those of UTs (0.24 mM for gulf toadfish *Opsanus beta*, 2.3 mM for *Desulfovibrio vulgaris*) (10, 12, 15, 24, 25). Thus, the affinities to urea of other types of UTs are all lower than that of the purified UrtA. Though *in vivo* affinity of UrtA has not been detected due to the difficulty in culturing *Prochlorococcus*, these results still suggested that UrtABCDE is a transporter better adapted to the low-concentration urea environment, which may confer the survival of *Prochlorococcus* in their natural environments with low concentrations of urea (14, 20).

**Table 1:** The affinity of different urea transporters to urea

*K*_d_ (nM)	*In vitro* experiment	*In vivo* experiment
UT	0.2 × 10^6^ (*Opsanus beta*) (25) 2.3 × 10^6^ (*Desulfovibrio vulgaris*) (10)	-
UreI	-	1.6 × 10^8^ (*Helicobacter pylori*) (23)
DUR3	3.0 × 10^3^ (*Arabidopsis thaliana*) (12) 2.2 × 10^4^ (*Zea mays*) (24) 3.2 × 10^4^ (*Paxillus involutus*) (15)	-
UrtA	14.0 × 10^3^ (*Saccharomyces cerevisiae*) (14)	141.3 (this study)

### Overall structure of UrtA/urea complex

**Figure 2:**
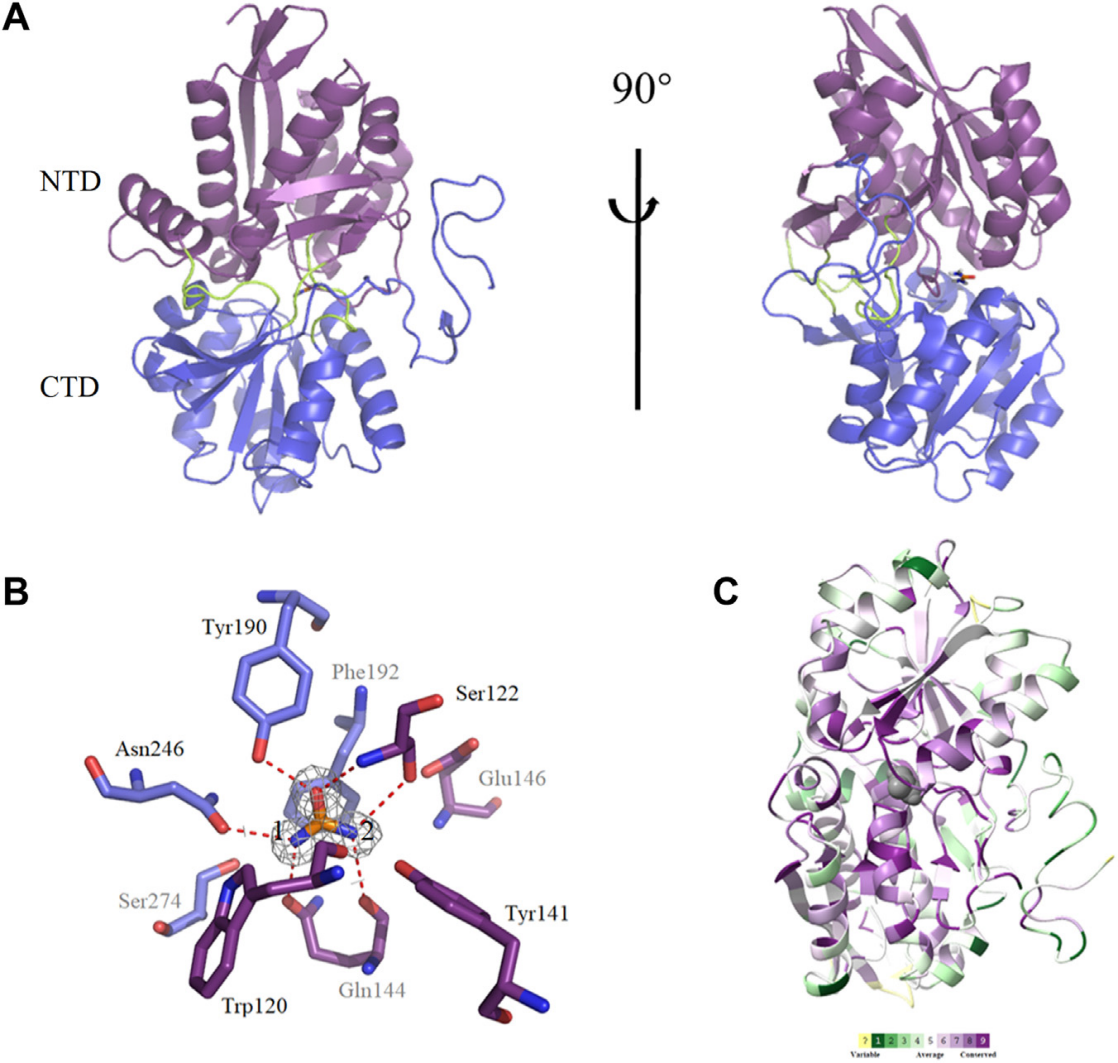
**Structure of the UrtA/urea complex.***A*, the overall structure of the UrtA/urea complex. The NTD is colored in *purple* and the CTD in *blue*; The NTD and the CTD are linked by a hinge region (*peak green*); the urea molecule is shown by *sticks*. *B*, the urea binding pocket of UrtA. The residues forming the binding pocket are shown by *sticks*. The 2*F*_*o*_*-F*_*c*_ densities for urea are contoured in *gray meshes* at 1.0 σ. Hydrogen bonds are lined by *red dash*. The two nitrogen atoms in the urea molecule are represented by the numbers 1 and 2. *C*, a ConSurf analysis of UrtA. The *color* scale indicates levels of conservation, 1 being low and 9 being high, with turquoise-through-maroon indicating variable-through-conserved. CTD, C-terminal domain; NTD, N-terminal domain.

In order to investigate the urea recognition and binding mechanism of UrtA, we attempted to crystallize the wildtype UrtA and UrtA complexed with urea. Finally, only crystals of UrtA complexed with urea were obtained. The crystal of UrtA/urea complex belongs to the P2_1_ space group and was solved to 1.60 Å (Table 2). The structure of UrtA/urea complex shows that each asymmetric unit contains one UrtA molecule. UrtA possesses the typical structural characteristics of cluster B-Ⅱ extracellular substrate-binding proteins. It consists of two domains, an N-terminal domain (NTD) and a C-terminal domain (CTD), linked by a hinge region (Fig. 2*A*). The NTD (residues 38–156 and 304–377) consists of eight parallel β-strands, which form a highly twisted β-sheet flanked on both faces by six α-helices. The CTD (residues 165–295 and 381–431) has a similar topology to the NTD, with a central core of seven parallel β-sheets flanked by six α-helices. The polypeptide chain crosses over three times in the middle of the two domains, forming a series of loop-shaped bends, which is the hinge region. The hinge region (residues157–164, 296–303 and 378–380) forms an interface, which contains the substrate-binding sites. The electron density map of the UrtA/urea complex structure shows that a single urea molecule is tightly bound to the hinge region between the two domains, interacting mainly with the residues in the hinge. Thus, the number of urea molecules bound in UrtA is consistent with our biochemical analysis above. By searching the Protein Data Bank, we found that the structures of the UrtA homologs from *Synechococcus* CC9311 (PDB code 7S6E, sharing ∼89% sequence identity) and *S.* WH8102 (PDB code 7S6F, sharing ∼92% sequence identity) have been deposited without further characterization. A structural comparison showed that the structure of UrtA from *P. marinus* MIT 9313 is almost exactly the same as UrtA from *S.* CC9311 and *S.* WH8102 (Fig. S1). The calculated RMSD between UrtA from *S.* CC9311 and *P. marinus* MIT 9313 is 0.190 Å based on 359 aligned atoms. The calculated RMSD between UrtA from *S.* WH8102 and *P. marinus* MIT 9313 is 0.180 Å based on 368 aligned atoms.

**Table 2:** Crystallographic data collection and refinement of UrtA/urea complex

Parameter	Value(s)^a^
	UrtA/urea complex
Diffraction data	
Space group	P2_1_2_1_2
Unit cell dimensions	
*a*, *b*, *c* (Å)	119.70, 47.61, 71.64
α, β, γ (°)	90.00, 90.00, 90.00
Resolution range (Å)	25.82–1.6 (1.66–1.60)
Redundancy	5.10 (5.30)
Completeness (%)	98.80 (99.39)
*R*merge^b^	0.09
*I/σI*	17.99 (4.06)
CC1/2	0.99 (0.88)
Refinement statistics	
*R* factor	0.16
Free *R* factor	0.19
Wilson B-factor	11.45
RMSD from ideal geometry	
Bond length (Å)	0.01
Bond angle (°)	0.83
Ramachandran plot (%)	
Favored	98.22
Allowed	1.78
Average B factor (Å^2^)	14.97

a Numbers in parentheses refer to data in the highest-resolution shell.

b *Rmerge=∑*_*hkl*_*∑*_*i*_*|I(hkl)*_*i*_*-<I(hkl)>|/∑*_*hkl*_*∑*_*i*_*<I(hkl)*_*i*_*>*, where *I* is the observed intensity, <*I*(*hkl*)>represents the average intensity, and *I*(*hkl*)_i_ represents the observed intensity of each unique reflection.

### Key residues of UrtA for urea recognition and binding

**Figure 3:**
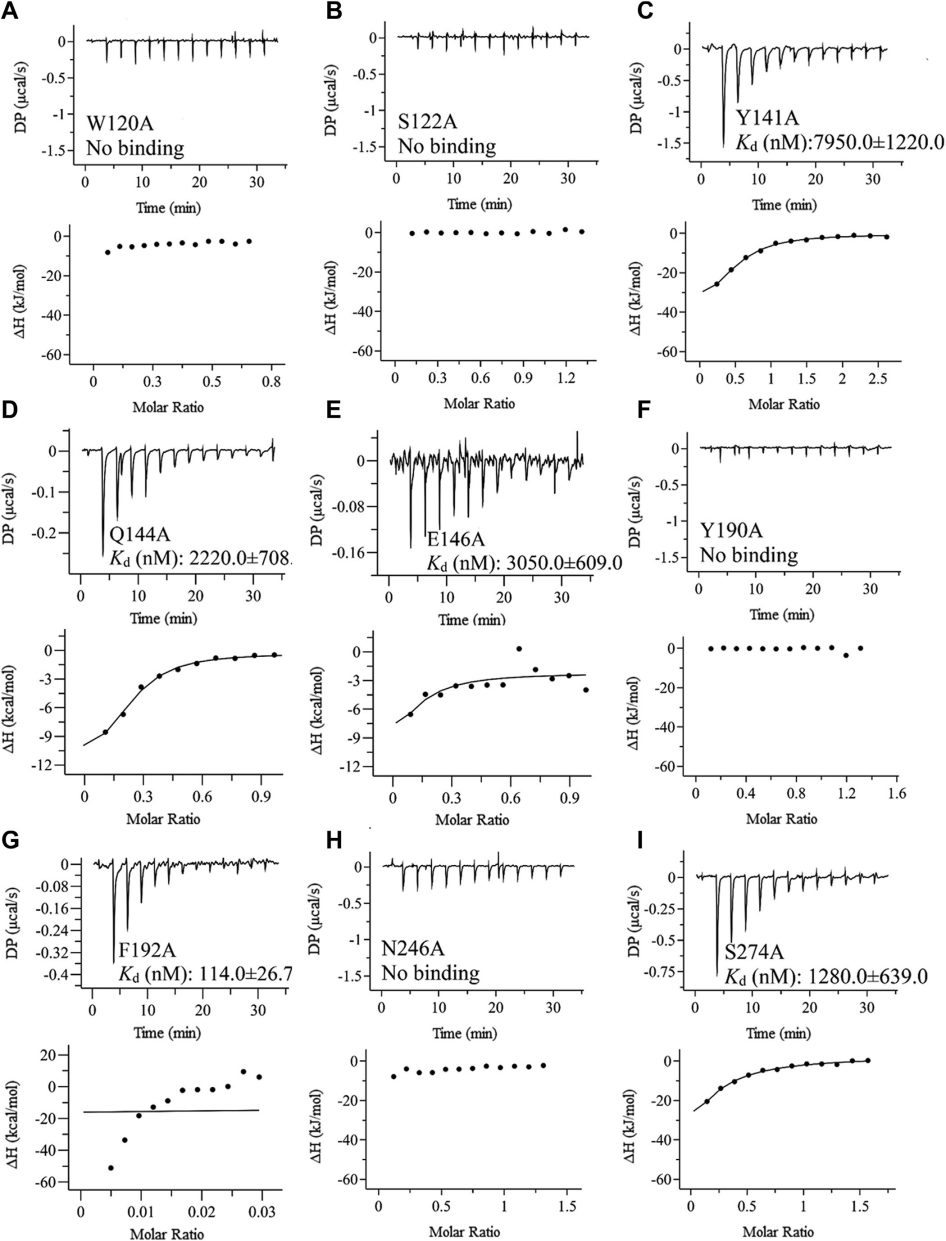
**The binding affinity of the mutants to urea.** ITC traces (*top*) and integrated binding isotherms (*bottom*) are shown. The thermodynamic parameter (Kd) of W120A (*A*), S122A (*B*), Y141A (*C*), Q144A (*D*), E146A (*E*), Y190A (*F*), P192A (*G*), N246A (*H*), and S274A (*I*) are shown in each ITC trace. The fitting error of one experiment is shown, whereas the results of the other experimental repeats are provided in Table S1. ITC, isothermal titration calorimetry.

**Figure 4:**
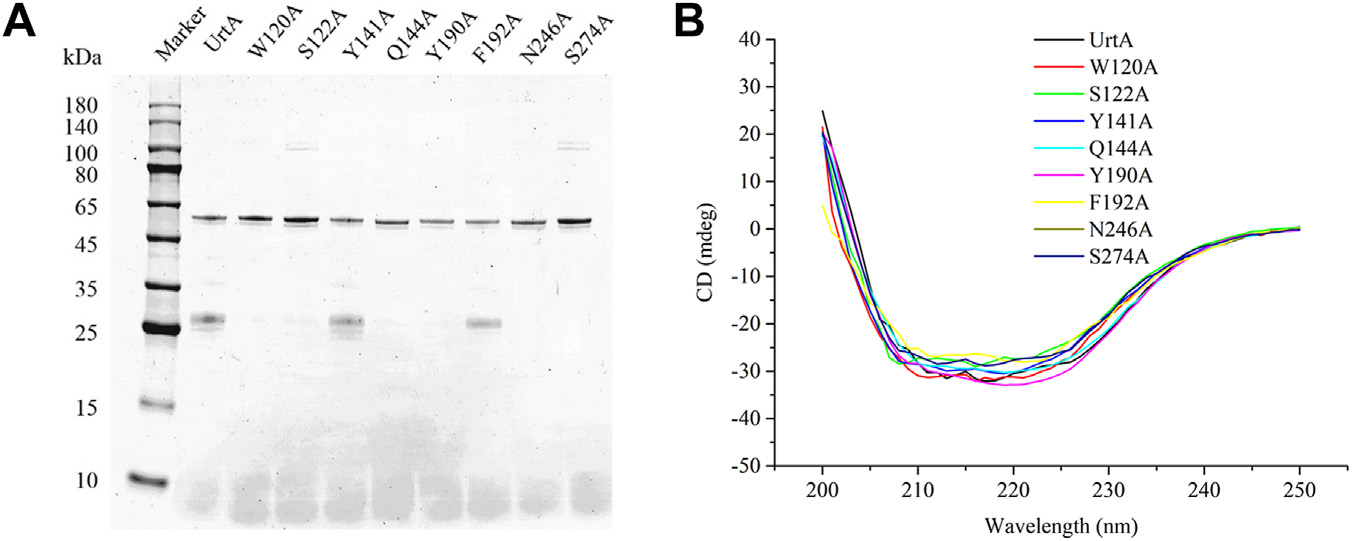
**SDS-PAGE analysis and CD spectra of the mutants of UrtA.***A*, SDS-PAGE analysis. *B*, CD spectra. CD, circular dichroism.

Structural analyses showed that nine residues (Trp120, Ser122, Tyr141, Gln144, Glu146, Tyr190, Phe192, Asn246, and Ser274) were close to the bound urea molecule (Fig. 2*B*). Conservation analysis showed that, except for Phe192, the other eight residues were highly conserved (Fig. S2). Six hydrogen bonds were observed. The first N atom of urea formed a hydrogen bond with the carbonyl group on the side chain of Asn246. The carbonyl groups on both the side chain and the main chain of Gln144 formed hydrogen bonds with the first and second N atoms of urea, respectively. The oxygen atom and the second N atom of urea were separately involved in hydrogen bonds with the amino on the main chain and the carbonyl group on the side chain of Ser122. The oxygen atom of urea and the hydroxyl group on the phenyl group of the side chain on Tyr190 formed a hydrogen bond (Fig. 2*B*). The six hydrogen bonds formed between urea and Ser122, Gln144, Tyr190, and Asn246 constituted a stable binding plane. Mutation on any of these four amino acid residues resulted in a significant decrease or complete loss of the urea binding affinity (Fig. 3, *B*, *D*, *F*, and *H*). In addition, we found that the mutant W120A was unable to bind to urea (Fig. 3*A*). However, the distance between Trp120 and urea was too large to facilitate noncovalent binding. The effect of Trp120 on urea binding may be attributed to the formation of hydrogen bond between the side chain of Trp120 and Asn246 (Fig. 2*B*), which stabilizes the position of Asn246, a key residue involved in urea binding. In contrast, mutations of Tyr141, Glu146, and Ser274 only resulted in a reduced binding capacity (Fig. 3, *C*, *E* and *I*). These residues are unable to form hydrogen bonds with urea but around the binding cavity. It is likely that mutations on these residues can alter the microenvironment and consequently affect the binding of urea. Different from the other eight residues, mutation of Phe192 showed little effect on the binding affinity (Fig. 3*G*), suggesting that Phe192 is not a key residue to stabilize the binding of urea. Circular dichroism (CD) spectra showed that the curves of all the mutants were similar to that of the wild-type UrtA (Fig. 4), suggesting that UrtA and the mutants have similar secondary structures and that the differences in the binding affinities resulted from residue replacement rather than secondary structure changes.

**Figure 5:**
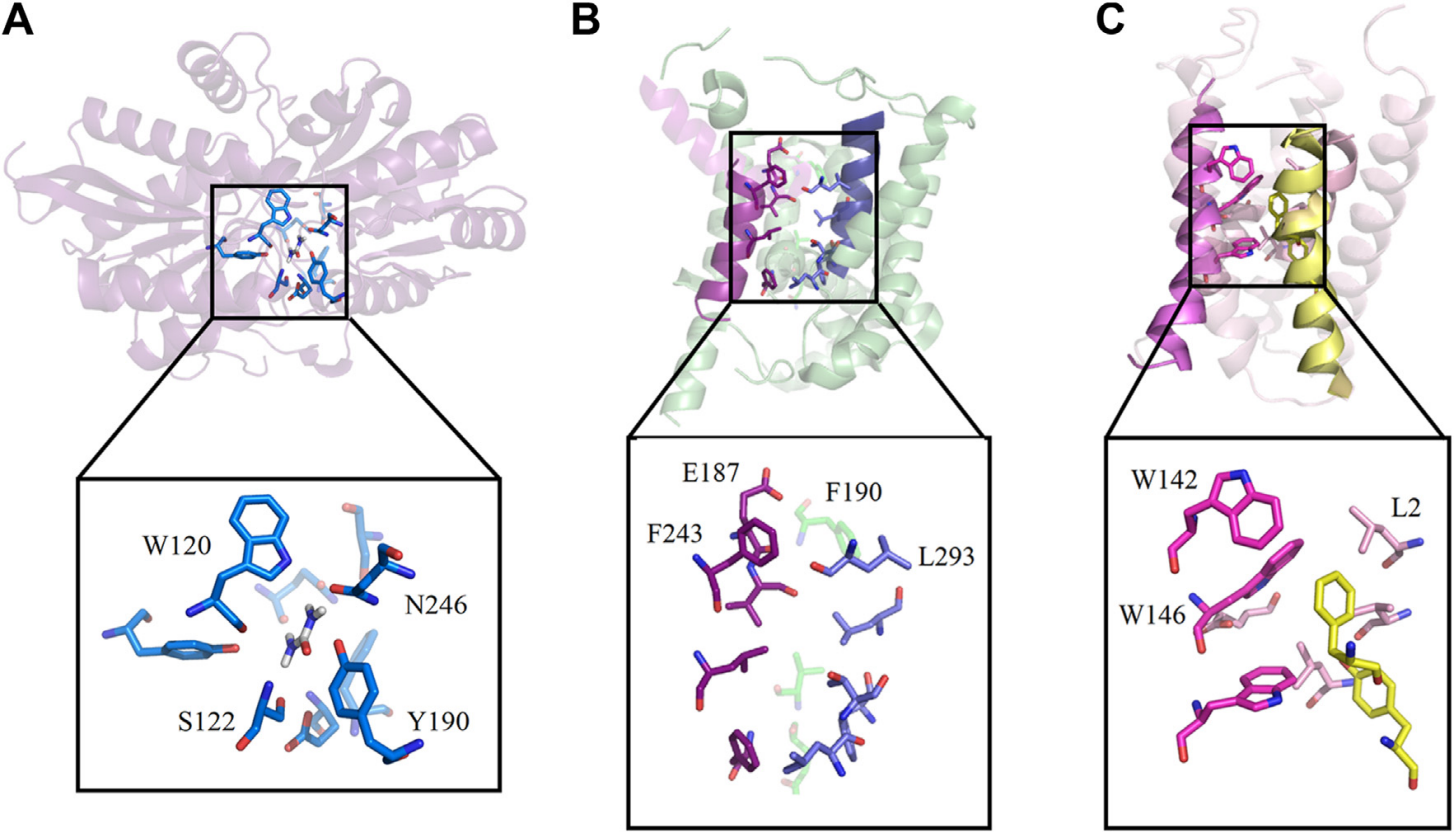
**Structural comparison of UrtA, UTs, and UreI.***A*, UrtA. *B*, UTs. *C*, UreI. Residues bound to urea molecules are showed by *sticks*, and the direction of urea transport is indicated by *arrows*. UTs, urea transporters.

In addition to UrtA, the structures of UT and UreI family were previously studied (10, 26, 27). Because of their different family origins, their mechanisms of recognition and binding of urea are quite different (Fig. 5). UT and UreI families do not have specific domains for initial urea recognition and binding. They all form urea transport channels and contain a selectivity filter for urea recognition. For the UTs (Fig. 5*B*), the carbonyl and side-chain oxygen atoms lining one side of the extracellular side region are responsible for initial urea recognition and binding (10, 28). For the UreI from *H. pylori* (Fig. 5*C*), the aromatic amino acid residues of a cavity in the extracellular structure can bind to urea molecules by forming hydrogen bonds (26, 27). Compared to other types of UTs, UrtABCDE shows a higher affinity for urea molecules. The specialized urea molecule recognition and binding domain, as well as strong hydrogen-bond interactions surrounding the bound urea molecule, are likely responsible for the high affinity.

### MDS of UrtA for urea recognition and binding

**Figure 6:**
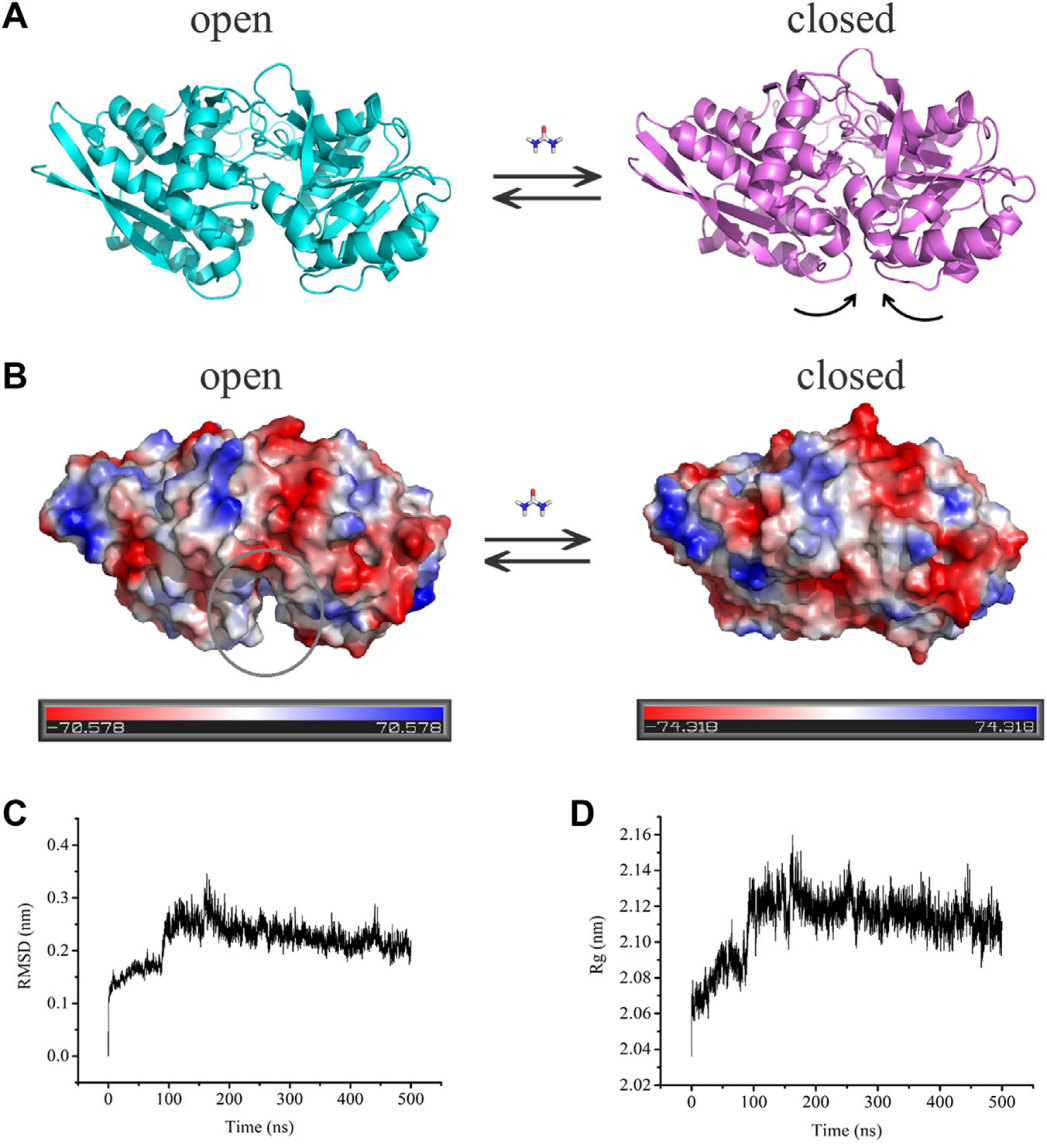
**Molecular dynamics simulation of UrtA.***A*, the open state and closed state of UrtA. The direction of the structural motion is indicated by *arrows*. *B*, electrostatic surface of open state and closed state of UrtA. *Red* denotes negative potential, and *blue* denotes positive potential. The opened tunnel in open state is indicated by *gray circles*. *C*, RMSF of the residues in the MDS of UrtA. *D*, Rg of UrtA. MDS, molecular dynamics simulation; RMSF, root mean-square fluctuation.

Sequence and structure analyses showed that UrtA is a cluster B-Ⅱ extracellular substrate-binding protein. Such proteins typically have the feature that the binding of the ligand will cause substrate-binding proteins to close around the ligand, much like a “Venus Flytrap” (29). In the UrtA/urea structure, the binding pocket was present in a closed state, and no entrance was found. Thus, we proposed that *apo* UrtA without binding urea contains a urea entrance and will undergo a similar closing process as other cluster B-Ⅱ proteins. To support this propose, we removed the bound urea molecule from the UrtA/urea complex structure and performed MDS for 500 ns. A simulated *apo* structure within an open state was then obtained (Fig. 6*A*). The RMSD profile of the backbone atoms of the simulated *apo* structure and the radius of gyration (Rg) of the enzyme molecule showed that simulation generated stable trajectories after 150 ns (Fig. 6, *C* and *D*), indicating that the system reached the equilibrium state. Following equilibrium, *apo* UrtA showed an open state (Movie S1), which is similar to the established open state of the bacterial periplasmic substrate-binding protein of methionine ABC transporter MetQ (PDB code: 6CVA) (Fig. S3) (21). In the steady state, an open hydrophilic entrance between the NTD and the CTD and near the hinge region was observed (Fig. 6*B*). Consistent with other cluster B-Ⅱ proteins (29, 30), the conformational change of UrtA without urea is like a “Venus Flytrap” (Fig. 6). These results suggested that UrtA undergoes a similar process to the typical cluster B-Ⅱ proteins, in which a urea molecule enters the binding site through the entrance aside the hinge region and causes *apo* UrtA to close around the urea molecule.

### The universality of the urea recognition and binding mechanism of UrtA in marine bacteria

**Figure 7:**
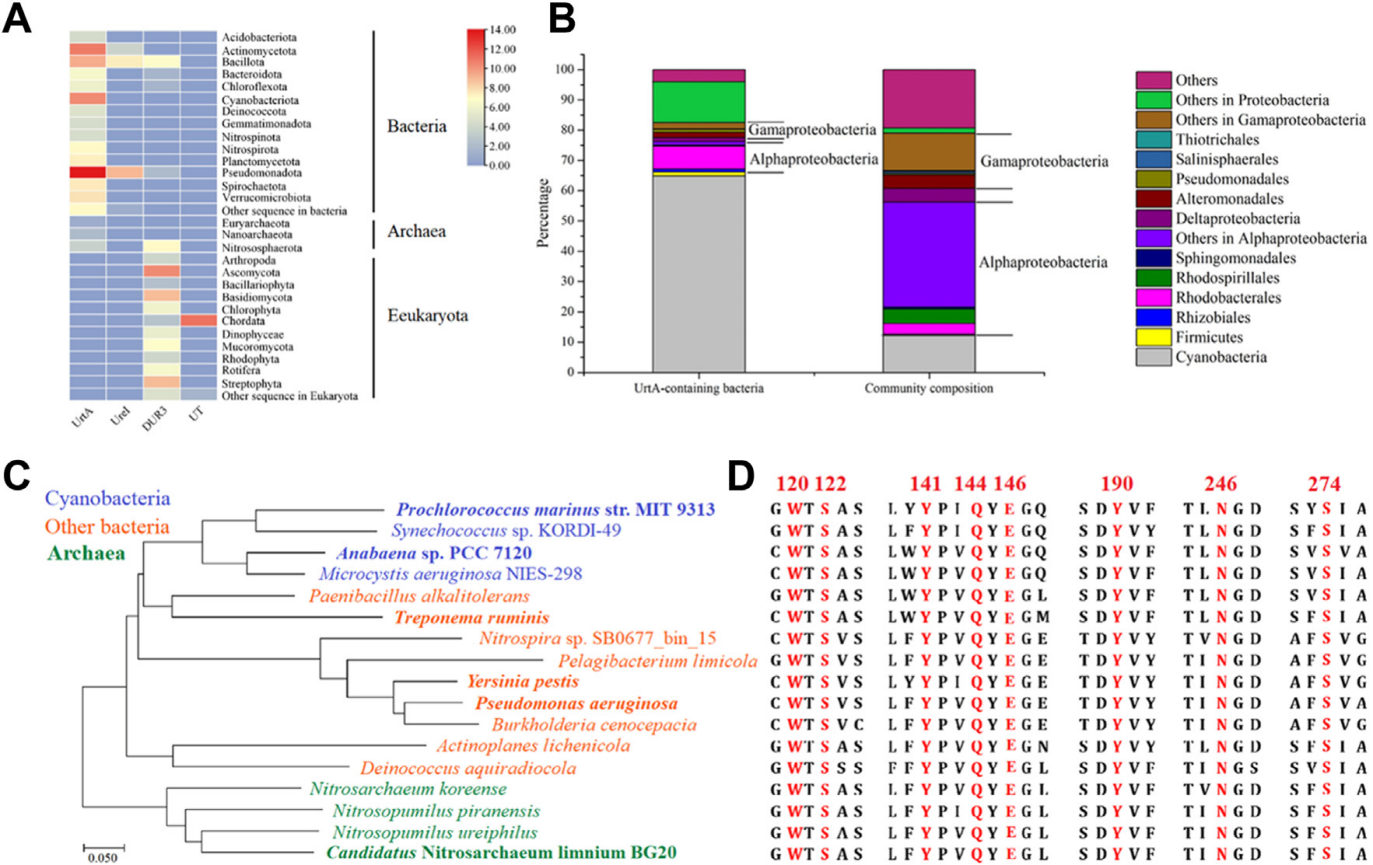
**The universality of the recognition and binding mechanism of UrtA in marine bacteria.***A*, relative abundance of uploaded sequence from different urea transporters at the phylum level. The heatmap was performed with TBtools (37). The values have undergone logarithmic processing, and the range of 1 to 14 corresponds to 2^n^, where n varies between 1 to 14. The *color* change from *pink* to *blue* represents the change from high abundance to low abundance. *B*, taxonomic compositions of bacteria containing UrtA and the microbial community retrieved from Tara Oceans database. The proportion of each taxon in the bacteria containing UrtA or in the microbial community is indicated by its percentage. *C*, evolutionary relationships of UrtAs from Archaea, and Bacteria. The evolutionary history was inferred using the neighbor-joining method. The bootstrap consensus tree inferred from 1000 replicates is taken to represent the evolutionary history of the taxa analyzed. Evolutionary analyses were conducted using MEGA7. *D*, sequence alignment of UrtAs from archaea, and bacteria in Figure 7*C*. The key residues in the binding pocket are colored in *red*. The alignment was performed with CLC Sequence Viewer 6.

Sequences from different families of UT were selected as queries to search in the Non-Redundant Protein Sequence Database. The screened sequences were classified by phylum (Fig. 7*A*). Sequences from the UreI family were predominantly found in *Proteobacteria*, with 97.9% of the retrieved sequences originating from *H. pylori*. Sequences from the DUR3 family were mainly found in *Ascomycota*, *Basidiomycota*, and *Streptophyta*. Sequences from the UT family were primarily found in *Chordata*. In contrast to UTs, UreIs, and DUR3s, sequences of ABC-type UTs are present in bacteria and archaea, particularly in *Actinomycetota*, *Bacillota*, *Cyanobacteria*, and *Proteobacteria*. For bacteria, the number of ABC-type UT sequences far surpasses that of other types of UTs. Moreover, except ABC-type transporters, no other UTs were found in *Cyanobacteria*, suggesting that *Cyanobacteria* may take up urea mainly by ABC-type transporters.

The distribution of UrtA homologs in marine bacteria was investigated by searching the Tara Oceans Database (Fig. 7*B*). The UrtA sequences were primarily found in the phylum *Cyanobacteria* (64.8%) and *Proteobacteria* (29.9%). In the microbial community of the Tara Oceans database, the proportions of *Cyanobacteria* and *Proteobacteria* were 12.2% and 68.4%, respectively. Among the phyla affiliated to *Proteobacteria*, *Alphaproteobacteria* was the most abundant group containing UrtA, accounting for 10.1%. The proportion of *Alphaproteobacteria* in the microbial community was 55.0%. *Gammaproteobacteria* was the second most abundant class harboring UrtA sequences, accounting for 5.0%. The proportion of *Gammaproteobacteria* in the microbial community in the Tara Oceans database was 18.3%. Thus, based on the data from the Tara Oceans database, marine bacteria carrying UrtA were mainly from *Cyanobacteria, Alphaproteobacteria* and *Gammaproteobacteria.*

Sequence alignment of UrtA homologs showed that the residues important for urea recognition and binding in UrtA, including Trp120, Ser122, Tyr141, Gln144, Glu146, Tyr190, Asn246, and Ser274, are strictly conserved in both archaea and bacteria (Fig. 7, *C* and *D*). Additionally, we randomly selected four UrtA homologs from bacteria and archaea (two of them near the closest matches to the lowest criteria) and detected the urea affinity of their recombinant proteins. The result showed that all of the proteins exhibited strong urea binding activity (Fig. S4). This result suggests that the screening criteria for searching in the Tara Oceans Database are reasonable and that UrtA homologs in bacteria and archaea likely utilize similar mechanisms for urea recognition and binding and have high affinity for urea like *Prochlorococcus*.

## Conclusion

*Prochlorococcus* is an important marine photosynthetic bacterial group, providing a huge amount of primary productivity in the ocean. It can grow in areas where N is limited. Urea is an important and efficient source of N for *Prochlorococcus*. However, molecular insight into the recognition of urea by *Prochlorococcus* is not known. In this study, we determined the function of the substrate-binding protein UrtA of the ABC-type UT from strain *P. marinus* MIT 9313, characterized its binding parameters to urea *in vitro*, solved its crystal structure, and investigated its mechanism for urea recognition. We found that of all proteins capable of transferring urea, the ABC-type UTs have the highest affinity to urea. Moreover, ABC-type UTs are widespread in marine bacteria, particularly in marine *Proteobacteria* and *Cyanobacteria*. The key residues associated with urea recognition and binding are highly conserved in UrtA from marine bacteria, suggesting that marine bacteria harboring ABC-type UTs may use similar mechanisms to recognize and bound urea. Our results provide a better understanding of urea transport in marine bacteria.

## Experimental procedures

### Gene synthesis and construction of point mutant

*urtA* (GenBank accession No. BX548175) without the predicted signal peptide from the marine bacterium *P. marinus* MIT 9313 was synthesized by the Beijing Genomics Institute. The gene was then subcloned into the pET-22b vector (TaKaRa) with a C-terminal 6× His tag. The resultant plasmid was named pET-22b-UrtA-W. All the point mutations were carried out by a QuikChange site-directed mutagenesis kit (Agilent) using the plasmid pET-22b-UrtA-W as the template.

### Protein expression and purification

The wildtype UrtA and its mutants were expressed in *E. coli* strain BL21 (DE3) (Vazyme). Cells were cultured at 37 °C in HB-PET autoinduction medium (Haibo) to an absorbance at 600 nm (*A*_600_) of 0.8 to 1.0 and then cultured at 18 °C for 32 h. Then, the cells were collected and lysed in the buffer containing 40 mM Tris-HCl (pH 8.0), 0.2 M NaCl, 5% (v/v) glycerol, 2 mM ethylene diamine tetraacetic acid, and 0.1 mM phenylmethanesulfonyl fluoride. The proteins were purified first with Ni^2+^-nitrilotriacetic acid resin (Qiagen) and then fractionated by gel filtration on a Superdex-75 column (GE Healthcare) in the buffer containing 20 mM Tris-HCl (pH 8.0), 200 mM NaCl, and 1% (v/v) glycerol. Ovalbumin (43 kDa) and conalbumin (75 kDa) from GE Healthcare were used as protein size standards to analyze the aggregative form of UrtA.

### Isothermal titration calorimetry

ITC measurement was performed at 25 °C using a MicroCal PEAQ-ITC system (Malvern). The sample cell was loaded with 300 μl of protein sample (20 μM), and the reference cell contained distilled water. The syringe was filled with 70 μl of urea solution (200 μM). The protein and urea solution were kept in the same buffer containing 20 mM Tris-HCl (pH 8.0) and 200 mM NaCl. Titrations were carried out by adding 0.4 μl of urea for the first injection and 1.5 μl for the following 12 injections, with stirring at 750 rpm. The measurement was repeated three times for each protein. The fitting error of only one experiment is shown in this article, and the results of other experiments are in Table S1.

### Crystallization and data collection

The purified UrtA was concentrated to approximately 5 mg/ml in 20 mM Tris-HCl (pH 8.0) containing 200 mM NaCl and 1% (v/v) glycerol. The UrtA protein mixed with urea at a molar ratio of 1:10 was crystallized at 18 °C by the hanging-drop method in the buffer containing 160 mM calcium chloride, 100 mM sodium acetate (pH 4.8), and 16% (w/v) polyethylene glycol (PEG) 6000. Glycerol (15%) was added to the crystallization buffer as a cryoprotectant. X-ray diffraction data were collected on the BEAMLINE BL19U1 at the Shanghai Synchrotron Radiation Facility using the detector DECTRIS PILATUS3 6M. The initial diffraction dataset was processed using the HKL2000 program. The statistics of the data collection are shown in Table 2.

### Structure determination, refinement, and conservation analysis

The phases were determined using the molecular replacement method (EPMR) and the CCP4 program Phaser (31). The crystal structure of UrtA/urea complex was solved using the crystal structure of AmiC (the controller of transcription antitermination in the amidase operon of *Pseudomonas aeruginosa*, sharing ∼29% sequence identity with UrtA), PDB code 1PEA, as the search model by molecular replacement. The structure refinement was performed using WinCoot and Phenix (32, 33). The quality of the final model is summarized in Table 2. All the structure figures were made using the PyMOL program (http://www.pymol.org/). Conservation analysis was performed using the online service ConSurf web tool (https://consurf.tau.ac.il/) (34).

### CD spectroscopic assay

UrtA and all the mutants at a concentration of approximately 10 μM in 20 mM Tris-HCl (pH 8.0) and 200 mM NaCl were subjected to CD spectroscopic assays at 25 °C on a J-810 spectropolarimeter (Jasco). CD spectra were collected from 250 nm to 200 nm at a scan speed of 500 nm/min with a bandwidth of 2 nm.

### Molecular dynamics simulation

The UrtA/urea structure was subjected to the software package GROMACS 2019 for a 500 ns MDS, with the amber99SB-ildn force field being adopted (35, 36). All simulations were performed under the NPT ensemble with periodic boundary conditions and a time step of 2 fs. The temperature of the system was kept at 298 K using the v-rescale method, and the pressure was kept at 1 bar using the Parrinello-Rahman method. According to the backbone-atom RMSD plot, trajectories that reached the equilibrium state (0–500 ns) were used for further analyses.

### Bioinformatics analysis

SignalP 5.0 was used to predict the signal peptide of UrtA. Four representative sequences from different families of UT (NCBI Reference Sequence: WP_011131593.1, WP_000901248.1, NP_199351.2, and XP_024839953.1) were selected as queries to search in the Non-Redundant Protein Sequence Database, with a stringent setting of an e-value cutoff < 1E-40, and percentage identity >40%. The screened sequences were classified by phylum. The full-length sequence (432 amino acid residues) of UrtA from *P. marinus* MIT 9313 was queried in the Tara Oceans database (http://ocean-microbiome.embl.de/companion.html) using tBLASTn to search for *urtA*-containing bacteria, with an E-value cutoff of 1E-40. The taxonomic composition of the microbial community was analyzed based on the 16S rRNA gene sequences from the Tara Oceans database. Evolutionary analyses were conducted using MEGA7. Multiple sequence alignment was performed and edited with CLC Sequence Viewer 6.

## Data availability

The atomic coordinates and structure factors of UrtA/urea complex have been deposited in the Protein Data Bank (PDB) under the accession code 8HIC.

## Supporting information

This article contains supporting information.

## Conflict of interest

The authors declare that they have no conflicts of interest with the contents of this article.
