# Supplementary material for: Structural and molecular basis for urea recognition by Prochlorococcus
Source: J Biol Chem. 2023 Jun 26;299(8):104958. doi: 10.1016/j.jbc.2023.104958 (PMC10392092; doi:10.1016/j.jbc.2023.104958)
Supplement: Supporting Information [file mmc1.docx]

**Supplementary Information for**

**Structural and molecular basis for urea recognition by *Prochlorococcus***


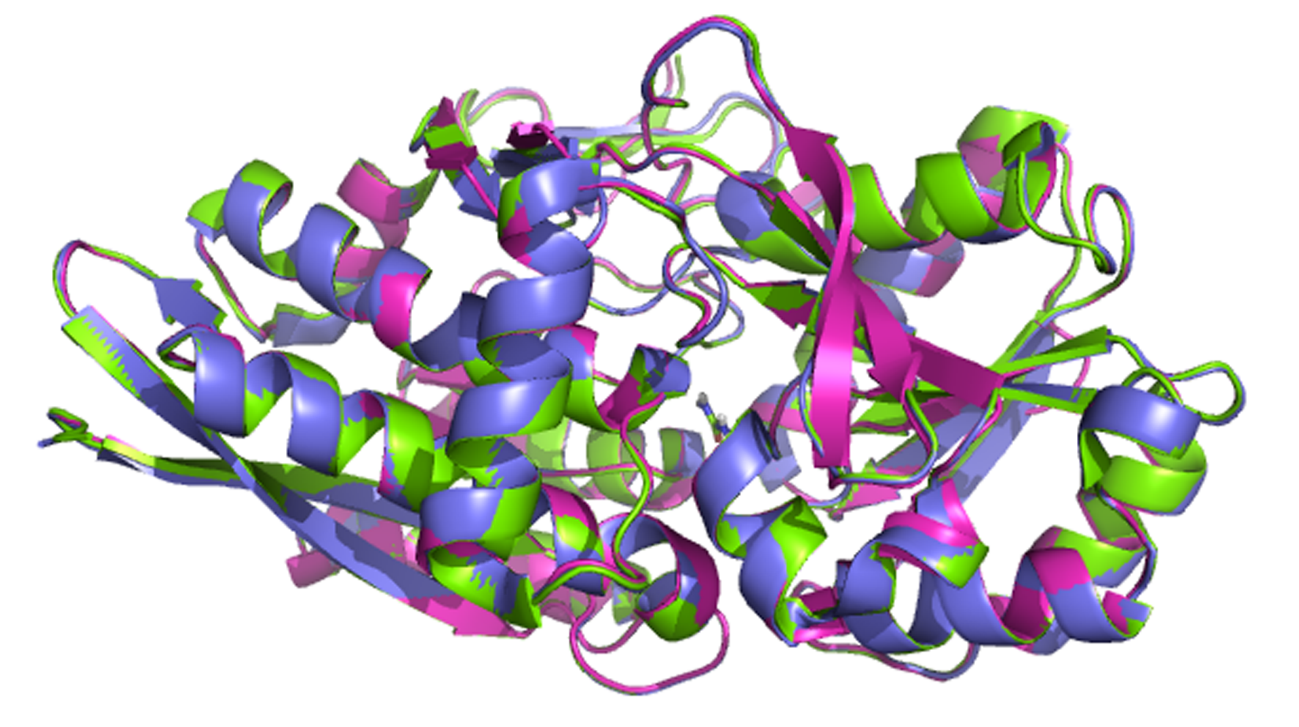
**Supplementary Figure 1. Overall structure of the UrtA /urea complex.** The structure of UrtA from *Synechococcus* CC9311 (PDB code 7S6E) is colored in chartreuse. The structure of UrtA from *S.* WH8102 (PDB code 7S6F) is colored in blue. The structure of UrtA from *P. marinus* MIT 9313 (PDB code 8HIC, this study) is colored in magenta. The urea molecule is shown by sticks.


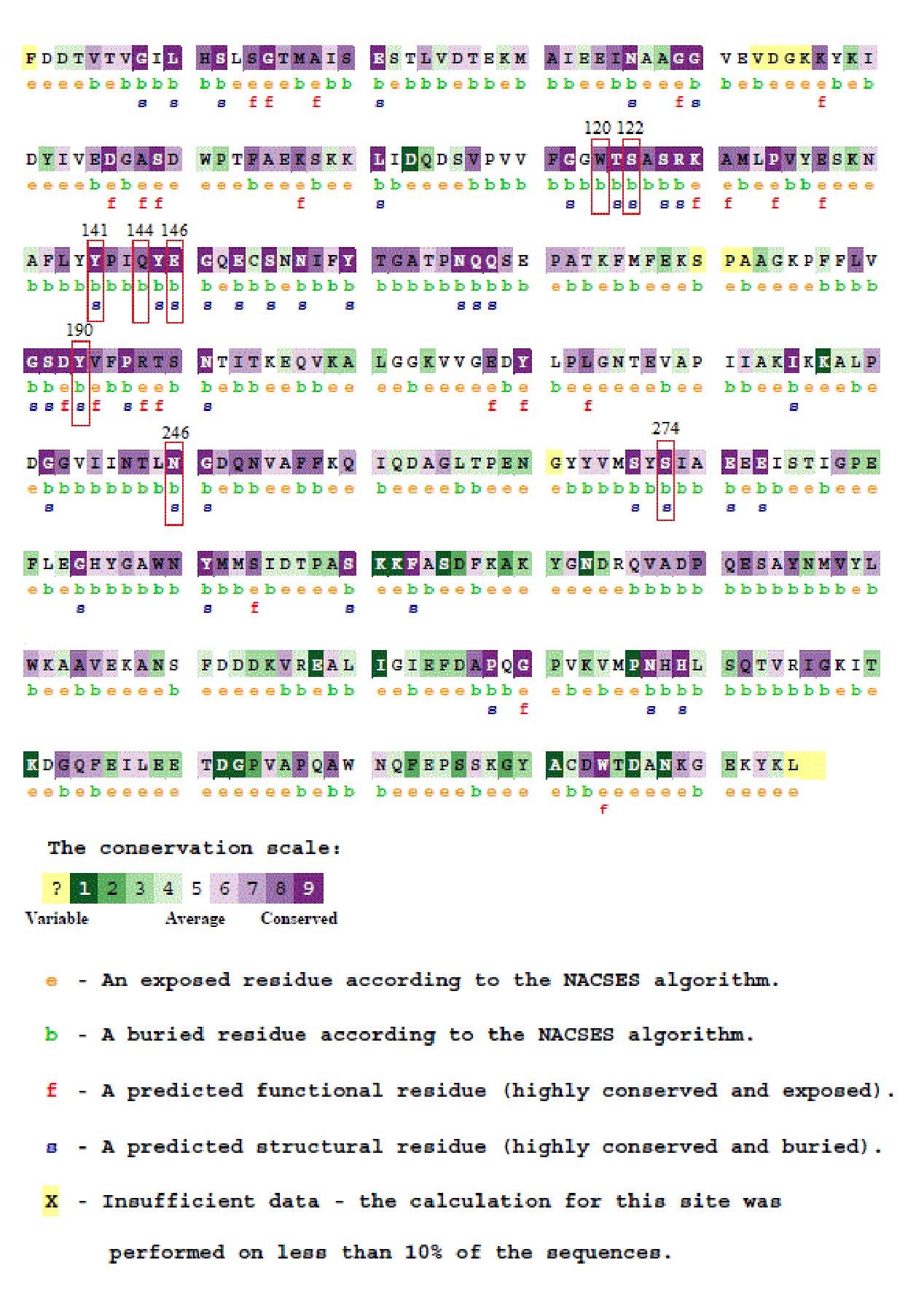
**Supplementary Figure 2. Plot displaying residual conservation in the UrtA sequence as generated by Consurf.** The color scale indicates levels of conservation, 1 being low and 9 being high, with turquoise-through-maroon indicating variable-through-conserved. The key amino acid residues in the binding pocket are marked by red box.


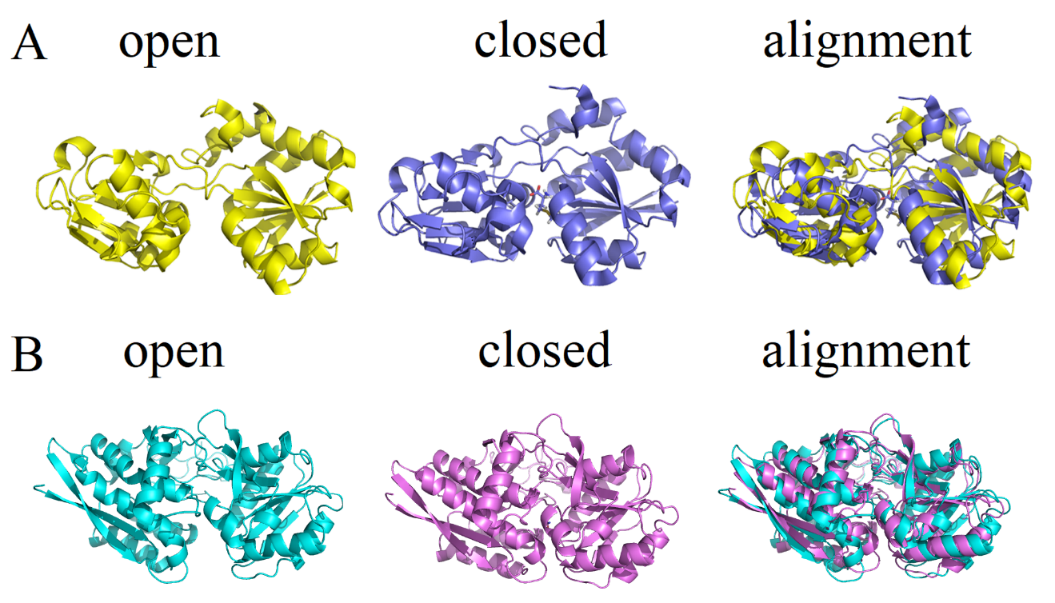


#### Supplementary Figure 3. The structure of MetQ (A) and UrtA (B). (A) The open state of MetQ (PDB code: 6CVA) is the crystal structure in substrate-free conformation. The closed state (PDB code: 6DZX) is the crystal structure in D-methionine bound conformation. (B) The open state and closed state of UrtA from *P. marinus* MIT 9313.

**
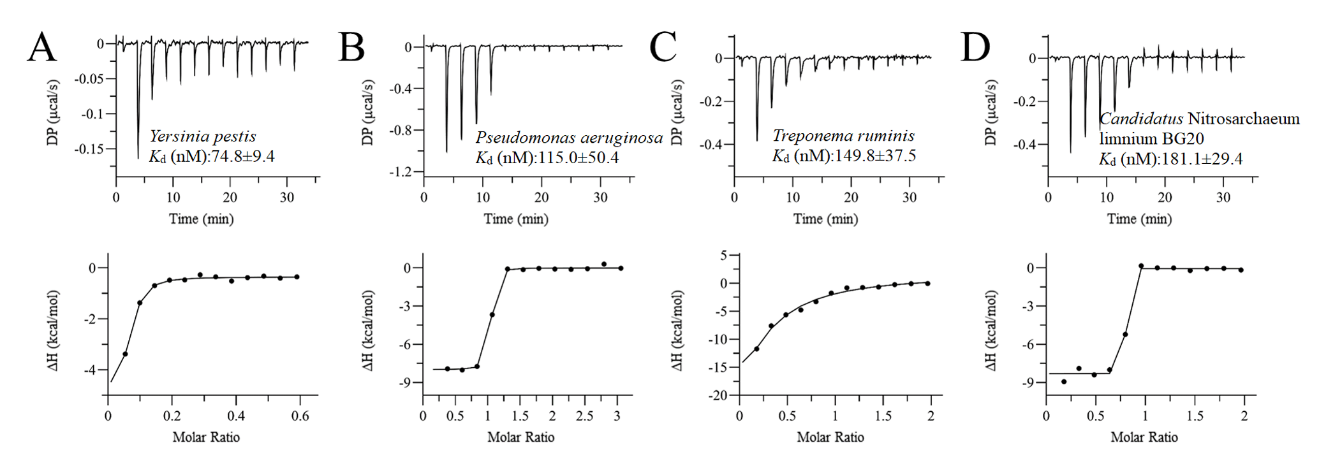
Supplementary Figure 4. ITC data for titrations of urea into UrtA from *Yersinia pestis*, *Pseudomonas aeruginosa*, *Treponema ruminis*, and *Candidatus Nitrosarchaeum limnium* BG20.**

**Supplementary Table 1. Thermodynamic parameters determined by ITC measurements**

|  | | N (sites) | *K*_d_ (nM) | *ΔH* (kJ/mol) | *ΔG* (kJ/mol) | *-TΔS* (kJ/mol) |
| --- | --- | --- | --- | --- | --- | --- |
| WT-1 | 0.718±0.007 | | 126.0±38.6 | -14.2±0.2 | -9.4 | 4.8 |
| WT-2 | 0.691±0.007 | | 141.3±11.2 | -13.4±0.3 | -9.7 | 3.7 |
| WT-3 | 0.528±0.004 | | 153.0±30.3 | -14.1±0.2 | -9.3 | 4.8 |
| W120A-1 | NB | | - | - | - |  |
| W120A-2 | NB | | - | - | - |  |
| W120A-3 | NB | | - | - | - |  |
| S122A-1 | NB | | - | - | - |  |
| S122A-2 | NB | | - | - | - |  |
| S122A-3 | NB | | - | - | - |  |
| Y141A-1 | 0.520±0.004 | | 7950.0±1220.0 | -10.5±1.5 | -7.0 | 3.5 |
| Y141A-2 | 0.520±0.043 | | 6470.0±1590.0 | -11.9±0.8 | -8.7 | 3.2 |
| Y141A-3 | 0.847±0.075 | | 1770.0±258.0 | -28±14.2 | -5.0 | 23 |
| Q144A-1 | 0.118±0.008 | | 2220.0±708.0 | -12.5±1.70 | -7.7 | 4.8 |
| Q144A-2 | 0.084±0.072 | | 8290.0±113.0 | -52.4±39.4 | -6.9 | 45.5 |
| Q144A-3 | 0.042±0.036 | | 8430.0±304.0 | -58.±10.3 | -6.9 | 51.1 |
| E146A-1 | 0.718±0.009 | | 3050.0±609.0 | -14.2±0.4 | -9.6 | 4.10 |
| E146A-2 | 0.691±0.009 | | 2350.0±1080.0 | -16.1±6.5 | -16.4 | -0.3 |
| E146A-3 | 0.528±0.006 | | 4330.0±420.0 | -58.9±11.0 | -16.4 | 42.5 |
| Y190A-1 | NB | | - | - | - |  |
| Y190A-2 | NB | | - | - | - |  |
| Y190A-3 | NB | | - | - | - |  |
| F192A-1 | 0.671±0.009 | | 114.0±26.7 | -79.9±0.0 | -5.9 | 74.1 |
| F192A-2 | 0.570±0.007 | | 103.3±8.1 | -80±14 | -9.5 | 70.5 |
| F192A-3 | 0.259±0.004 | | 95.4±7.9 | -60.9±12.5 | -9.5 | 51.3 |
| N246A-1 | NB | | - | - | - |  |
| N246A-2 | NB | | - | - | - |  |
| N246A-3 | NB | | - | - | - |  |
| S274A-1 | 0.313±0.006 | | 1280.0±639.0 | -80.0±0.4 | -6.6 | 73.4 |
| S274A-2 | 0.368±0.008 | | 1410.3±100.7 | -80.4±14.9 | -6.7 | 74.4 |
| S274A-3 | 0.202±0.001 | | 1440.0±106.5 | -75.6±5.5 | -5.5 | 70.2 |

NB: no binding. *ΔH*, change in enthalpy; *T*, temperature; *ΔS*, change in entropy; *ΔG*, change in Gibbs free energy.

**Sequences of the selected UrtA homologs:**

>NSL64327.1 urea ABC transporter substrate-binding protein [*Yersinia pestis*]

MQRRNLIKVFALSASVISMGMAFGTLAADTIKVGILHSLSGTMAISETPLKDMALMSIDDINAHGGVLGKQLEPVIVDPASNWPLFAEKARQLLTQDNVAVVFGGWTSVSRKSVLPVFEELNGLLFYPVQYEGEEMSPNVFYTGAAPNQQAIPAVEYLLSEEGGAAKRFILLGTDYVYPRTTNKILRAFLHTKGIEDKDIEEVYTPFGYSDYQTIVGNIKKFSAGGKTAVISTINGDSNVPFYKELANQGIKATDVPVIAFSVGEEELRGIDTKPLVGNLAAWNYFQSVDNPTNKQFVEHWKAYAKANKLPNADSAVTNDPMEATWVGMHMWAQAVEKAQSTDVDKVRAAMAGQTYAAPSGFTLTMDQTNHHLHKPVMIGEIEADGQFNVVWQTDAPIRAQPWSPFIAGNDKKPDHPVKAASSHQIANNSRQMIAVNR

>WP_124014657.1 urea ABC transporter substrate-binding protein [*Pseudomonas aeruginosa*]

MHNDTYRLIKRALFLGVFSLLPLALVSQAQAAETVKVGIIHSLTGTMAMSEASVVDAEKLAIDEINASGGVLGKTIETIVEDGASDWPTFAEKARKLLESDRVVVTFGGHTSASRKAMLPVFERNKGLLYYPTFYEGLEKSPAIIYTGAEASQQTLAAVGWLMANKGKSVYLIGSDYIWPRTTNKLARASVSKHGGSIVGEDYLPLGSIEFSSVINKIKAAKPDIVLSTVVGGSNVAFYKQLKAAGIDSSNQTLMALAVTEEEVTGIGAENLTGFLTCMSYFQSLENPVNEKFVAAFKARYGDNRVVGDPMAAAYTAVYLWKKAVEKAGSFDVPAVIAASSELTLDAPEGEVKVHKDNHHLWKRARIGAINAQGQVDVIYESAPIEPNPFPKL

>QSI01380.1 urea ABC transporter substrate-binding protein [*Treponema ruminis*]

MNMKKVTMGIAGIALSALLLASCNQKKPETVKVGLLHSLSGTMSISETAVRDAELLAISEINENGGVLGRQIEAIQADGASEPQVFAKKARELLQDEKVVTVFGCWTSASRKAVKPIFEELYGLLWYPVQYEGMEASPNIMYMGASPNQQIVPAVDYCAKNIGKKMFLVGSDYVFPRTANKIIKAQIAQLDGECAGEEYLPMGATNFSEVVAKILATKPDVILNSLNGDSNVSFFAALAKAGITSSTIPVMSFSIAEEEVAKMDLDNLAGHLVSWNYYETTQTPRNEKFVSDYKEKYGEDRMTGDPIEAAYIAVYMWAAACEKAGGFEVEAVRVAAKGLSFTAPEGTVTIDGGNQHLYKQVRIGKINNKGLIDEVWATPSAVKPDPYLSTYPWARGL

>EPA05038.1 urea ABC transporter, urea binding protein [*Candidatus Nitrosarchaeum limnium* BG20]

MFLSVQNGYLNYSSSERKTIIEKPSMILNKENPIKVGILHSLTGTMAISETAVADATLMAIEEINDRGGILGREVVPILKDGKSDWSIFALEAKNLIVEDKVSVVFGGWTSASRKTMKPVFEEYDHLLFYPIQYEGLEKSPNIIYTGASPNQQVLPAIDWAYENLGPRFFLVGSDYVFPRSVNEIIKGKVHELGGVVVGEEYKLLGEENFKDVVDKIVESKPDVILNTINGDSNCSFFNELRQRGITPKVIPTISFSIAEDEIKIIGAEKMAGDYAAWNYFQSLDNKYNNDFVKNFKKKYGEDRVVDDPMESGYVGVYLYAKAVAMAGTDDISIVREKLKGLTFHAPEGAVGIDPQNQHLTKVVRIGQILPDGQFKIVSSSENQIKPNPFPDYKTEEQWNEFLDNLYKGWNENWANPGTVSVIQK
